# Detection of Illicit Conservation Treatments in Sea Bass (*Dicentrarchus labrax*): Application and Data Integration of NIR Spectrometers

**DOI:** 10.3390/foods13213443

**Published:** 2024-10-28

**Authors:** Giovanna Esposito, Alessandro Benedetto, Elisa Robotti, Masho Hilawie Belay, Eleonora Goggi, Simone Cerruti, Nunzia Giaccio, Davide Mugetti, Emilio Marengo, Laura Piscopo, Marzia Pezzolato, Elena Bozzetta, Maria Cesarina Abete, Paola Brizio

**Affiliations:** 1 Istituto Zooprofilattico Sperimentale del Piemonte, Liguria e Valle d’Aosta, Via Bologna 148, 10154 Torino, Italy; giovanna.esposito@izsto.it (G.E.); nunzia.giaccio@izsto.it (N.G.); davide.mugetti@izsto.it (D.M.); marzia.pezzolato@izsto.it (M.P.); elena.bozzetta@izsto.it (E.B.); mariacesarina.abete@izsto.it (M.C.A.); paola.brizio@izsto.it (P.B.); 2 Department of Sciences and Technological Innovation, University of Piemonte Orientale, Viale Michel 11, 15121 Alessandria, Italy; masho.belay@uniupo.it (M.H.B.); ele.goggi@gmail.com (E.G.); simone.cerruti@uniupo.it (S.C.); emilio.marengo@uniupo.it (E.M.); laura.piscopo@uniupo.it (L.P.)

**Keywords:** Cafodos, sea bass, food fraud, near infrared spectroscopy, chemometrics, PLS-DA

## Abstract

Global fish and seafood consumption is increasing annually, frequently leading to the emergence of food fraud, mainly related to mislabeling and adulteration like, for example, the use of illicit/unauthorized food additives to mask or delay fish spoilage. Among the available diagnostic tools for control purposes, spectroscopic techniques have often been proposed to identify these kinds of illicit practices in fish and seafood products. The presented study aims to test two cheap and portable near infrared (NIR) spectrometers, a handheld MicroNIR and a pocket-sized SCiO, to uncover use of the illicit food additive Cafodos, a mixture of sodium citrate and hydrogen peroxide used to preserve some fish characteristics (like smell, color, na dtexture). The NIR spectroscopy in combination with chemometric approaches, allowed the successfully classification of (81–100%) samples of sea bass (*Dicentrarchus labrax*) treated with Cafodos. The study highlights the potential of this technique that, by not requiring pre-treatment of samples with further reagents, is cheaper and safer for the environment. In conclusion, the study confirmed the potential of portable devices for rapid NIR spectroscopy analysis to identify food fraud and ensure consumer safety.

## 1. Introduction

In the seafood industry, fraud, defined as “the intentional act of substituting, adding, adulterating, tampering with, or misrepresenting illicit ingredients or treatments”, represents a primary threat to the production and marketing of seafood products, especially regarding widely consumed species [1]. These frauds are rising as a result of the market’s need to meet the consumer’s demand, especially for fresh fishery products, characterized by short shelf life and, therefore, are often prone to be treated and/or manipulated to mask and reduce spoilage [2].

Seafood freshness is indeed a key quality parameter due to the short shelf life of this highly perishable foodstuff in relation to safety, nutritional value, availability, and edibility [3,4]. This quality declines rapidly after capture. Therefore, critical control processes in the supply chain, like adequate instant processing, transport, packaging, and storage are needed in order to preserve nutritional properties and avoid safety risks. To maintain the organoleptic characteristics or mask the actual state of aging, numerous treatments have been developed, and are sometimes illicit or dangerous for consumers [5]. Among these, there are Cafodos or hydrogen peroxide [6,7], chemical products used illegally as additives to preserve the freshness characteristics of the fish, which thus makes it appear apparently alive. The Cafodos additive, however, is difficult to find in the treated product because once it is in contact with water and ice, it melts and all traces of it are rapidly lost. By subtly conferring an appearance of freshness to the fish, the use of Cafodos constitutes both a commercial fraud, i.e., the sale of a stale or altered fish for a fresh and sanitary one, since the food apparently seems to retain the characteristics of freshness, while internally it undergoes deterioration, giving rise to previously cited allergic and/or poisoning problems in humans like scombroid syndrome [2].

Sea bass, (*Dicentrarchus labrax*) is one of the most important fish species from an economic point of view in the entire Mediterranean area; Turkey and Greece have recently become the major producing countries, while Italy, Spain, and France continue to remain the main world importers [8].

To the best of our knowledge, the frauds most detected for this fish species are those on the mislabeling: incorrect declaration of origin of farmed/wild fish and correct fresh/frozen status of fish [9,10,11,12]. Only few studies are available on the rapid discrimination of fish samples treated with compounds to simulate freshness [13].

To prevent this circumstance and face the threat of the marketing of spoiled or adulterated products, it is essential to find new technologies, that are rapid and in step with the times, to identify illicit treatments in real-time, to report them to the authorities as an alternative to the total volatile basic nitrogen (TVB-N), the main chemical indicator for spoilage assessment in seafood products [14]. Untargeted fingerprinting approaches based on Raman or near infrared spectroscopy (NIRS) meet all these characteristics, as they provide multiple chemical and physical information to qualitatively/quantitatively characterize complex food matrix [15]. In a previous study, the effects of Cafodos were studied on sea bream with Raman spectroscopy highlighting its capabilities to discriminate between treated and not treated samples with good accuracy [16]. Following this study, we decided to use the same protocol proposed by Benedetto et al. (2024) [13] to test the efficacy of NIR spectroscopy on sea bass to evaluate illicit treatment with Cafodos. The shape of the NIR spectra obtained from fish samples is the result of several interactions between NIR radiation and water; organic molecules like protein, carbohydrates, and fat; and low-concentration constituents such as vitamins and minerals [17]. Indeed, NIR analysis supported by chemometric techniques has been emerging as an effective tool in food fraud analysis [18,19], as it is proven to be able to detect alterations caused by different kinds of fish processing [20]. In addition, the use of portable NIR instruments has been often adopted in several applications [21].

The purpose of this project was to evaluate the possibility of discriminating sea bass illicitly treated with Cafodos from untreated ones using spectral acquisitions from NIR portable devices coupled with multivariate analysis. The instruments used in this study have different portability and spectral measurement ranges. In detail, the following were used: a portable device controlled by a smartphone (SCiO by Consumer Physics, Tel Aviv, Israel), and a portable MicroNIR instrument (Viavi Solutions Inc., Santa Rosa, CA, USA) controlled with a laptop PC.

## 2. Materials and Methods

### 2.1. Reagent and Samples

Hydrogen peroxide solution (≥30%, for trace analysis) and sodium citrate tribasic dihydrate (≥99.0%) were purchased from Merck Life Sciences (Milan, Italy). A Cafodos-like treatment solution (TS) composed of 8 g/L hydrogen peroxide and 2.5% (*w*/*v*) sodium citrate was prepared in ultrapure water and stored in a refrigerator at 4 °C.

Fish samples were purchased from a single farm soon after capture, through a trusted supplier chain able to deliver animals in less than 24 h after capture. Fish were, therefore, homogeneous in size (600 g average gutted weight) and production cycle. A total of 24 farmed European sea bass specimens (*Dicentrarchus labrax*) collected during spring 2023 were used. Samples were divided into four groups: controls (stored on ice in the fridge for 3 h and for 24 h, respectively) and a group treated with a Cafodos solution for 3 h and 24 h, respectively. For more details about the study design, refer to [13,16]. The experimentation was carried out over 12 different days, with two samples evaluated and treated as paired comparisons each day: one control and one treated fish.

Each treated fish was placed in the Cafodos solution for 60 s, transferred into a food bag and stored on ice in the fridge for 3 h or 24 h (ratio fish:solution 1:1 *w*/*v*). The corresponding control samples were treated and stored in the same way, by using ultrapure water. Each fish was washed by flushing it with deionized water for 2 min, soon after treatment.

Spectroscopic measurements were taken from different fish parts as follows: eye, gills, skin, and muscle.

Samples were labeled with the following 9-digit string: a letter indicating the sampling site (E = eye, G = gill, M = muscle; S = skin); the indication of the fish sample (“F” followed by a two-digits progressive number); the indication of the group (“C” for controls and “T” for treated samples); a 2-digit number indicating the short- or long-term (03 = 3 h; 24 = 24 h); a 3-digit string indicating the replication (“_” followed by a progressive number). These sample labels were the same as those exploited in a previous paper where Raman spectroscopy was tested on the same sample batches [16].

### 2.2. NIR Acquisition

NIR spectra were acquired directly on the skin, eye, muscle, and gill of each sample. Three replications of each measurement were recorded; for muscle and skin, they were obtained from three different positions of the fish’s middle back, while for the gills and eye, they were obtained by re-positioning the instrument on the area each time. The study was conducted using 2 portable NIR spectrometers (SCiO pocket device, by Consumer Physics, and MicroNIR 1700 Pro ES VIAVI Solutions, San Jose, CA, USA).

#### 2.2.1. Portable SCiO

Spectra NIR collection and management of data were performed using the pocket-sized SCiO device with a probing size of 0.6 × 0.9 mm and connected to a smartphone app (The Lab, v. 1.3.1.81). Spectra were acquired in diffuse reflection mode. The experimental acquisition parameters were set as follows: 740–1070 nm as the spectral range, 10 cm^−1^ resolution, with a typical scan time of less than 5 s.

Each collected NIR spectrum was stored on the online Consumer Physics cloud database. The SCiO sensor was calibrated before the first acquisition by placing the sensor in the instrument lid and performing a scan. The lid housing serves as a reference and blank. A new calibration was performed when notification appears.

#### 2.2.2. Handheld MicroNIR

Spectra NIR collection and management of data were performed using the handheld MicroNIR with a probing size of 47 mm diameter. Analytical conditions for the MicroNIR were as follows: 908–1676 nm spectral range, 12 cm−1 resolution, 12.5 µs integration time, and 200 scans at 80 Hz were applied. The spectra acquisition and instrumental control were managed by means of the VIAVI MicroNIR Pro software (v2.0), operated on a laptop computer. Before the first sample, a dark current acquisition was performed followed by a reference standard acquisition to calibrate the instrument.

### 2.3. Data Analysis

For both the SCiO sensor and the micro-NIR measurements, multivariate statistical methods were applied to provide classification models able to classify the samples as control or treated and identify the differences between the classes (controls vs. treated samples).

For this purpose, in the case of the SCiO, the collected spectra, uploaded and stored on Consumer Physics’ cloud database, underwent the following approach: (i) spectra were subjected to the four owners pre-treatments algorithms: Processed”, “Normalize”, “Processed & Normalize”, and “(log)R & Normalize” which included logarithmic, first and second derivative, mean average scan, and SNV transformation; (ii) a principal component analysis (PCA) was used as an unsupervised approach to verify the possible grouping of the samples according to the treatment; (iii) random forest (RF) was applied as a classification method for the identification of the differences between the control and treated samples. RF and cross-validation were automatically performed by the app, in fact, the algorithms are proprietary and it is not possible to change the set-up or obtain further information regarding the chemometric processing of the spectra.

In the case of micro-NIR measurements, instead, the collected spectra underwent a different approach as follows: (i) spectra were first pre-treated by different procedures (SNV, first and second derivative); (ii) classification was performed by partial least squares–discriminant analysis (PLS-DA) coupled to a variable selection procedure in backward elimination (BE) to identify the differences between the control and treated samples and provide the best predictive ability. In particular, the Savitzky–Golay procedure was applied for the calculation of first and second derivative spectra with a step 5 and using a second order polynomial. BE-PLS-DA [13,16] was then applied to provide the classification of the samples (control vs. treated) separately for the short- and long-term exposure. PLS-DA is based on partial least squares (PLS), a multivariate regression method able to correlate X (the descriptors, here the wavelengths) and Y variables (the responses), by identifying couples of latent variables (LVs) on X and Y blocks that mostly correlate. PLS-DA is the classification version of PLS, where the Y variable represents the class membership. Here, PLS-DA was applied to autoscaled data, after pretreatment by SNV and first or second derivative. The algorithm was also coupled to an iterative variable selection procedure in backward elimination; at each iteration, the variables with the smallest VIP score [22] in cross-validation, were eliminated (no more than 6% of the variables were eliminated at each cycle). Leave-more out cross-validation was applied here, taking into account that the study was designed as paired comparisons. During cross-validation, all the replications of the measurements of the fish samples that were analyzed in the same day were taken out contemporarily (6 cancellation groups). The work presented here is part of a greater project aimed at characterizing the applied treatments by different spectroscopic tools [16] and a multi-omic approach [13]. The study design was, therefore, aimed at finding the best compromise between: (i) a proper evaluation of the individual variability; (ii) the minimization of the experimental effort; (iii) the characterization of the samples in a short time after treatment. For these reasons, the calculations were applied in cross-validation and no test set was applied; however, the applied cross-validation procedure provides an evaluation of the predictive ability of the models that can be considered reliable enough for a first feasibility study.

The classification results were evaluated on the basis of parameters related to the overall classification performances (% accuracy and non-error rate—NER%).

For the micro-NIR calculations, spectra pre-treatment and classification models were calculated exploiting self-developed routines in Matlab R2014b (The Mathworks, Natick, MA, USA) and the Classification Toolbox from the Milano Chemometrics group [23]. Figures and plots were carried out by Statistica v7 (Statsoft Inc, Tulsa, OK, USA).

## 3. Results

### 3.1. SCiO

The acquisitions on the eye were not saved because the SCiO instrument reported an error in the intensity of the recorded signal and, therefore, the eye was not taken into consideration.

The first explorative analysis to evaluate the behavior of fish samples was the principal component analysis (PCA); samples were not effectively grouped according to the treatment applied. Subsequently, the four algorithms for data pre-treatment presented in the app were used before the RF classification models and compared in terms of coefficient of variation (F1) and confusion matrix to evaluate which algorithm provided the best classification results.

The ”F1” corresponds to the performance of the model, and “1” corresponds to the best classification.

**Figure 1:**
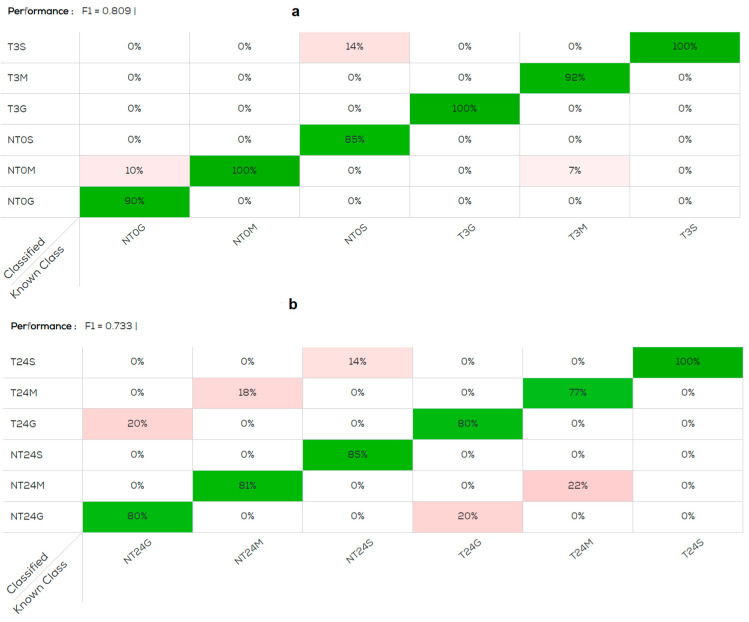
Confusion matrix obtained with model normalised, pre-treatments: average scans and SNV (standard normal variate). (**a**) Gill (G), muscle (M), skin (S). NT0G; NT0M; NT0S: not treated; T3G; T3M; T3S: treated and acquired post 3 h; (**b**) NT24G; NT24M; NT24S: not treated; T24G; T24M; T24S: treated and acquired post 24 h. The percentage of samples correctly classified by category is represented in green, the percentage of samples classified in an incorrect category is represented in pink.

The best data obtained from all organs at 3 and 24 h are reported in Table 1 for the data pre-treated by average scans and SNV. The performance of the confusion matrix obtained by analyzing all treated and untreated organs at 3 h (Figure 1a,b) is better, F1 = 0.809 than that obtained at 24 h, F1 = 0.733. In both confusion matrices, the skin of the treated fish was the organ with 100% correct classification, confirming that the Cafodos modifies the fish appearance. Subsequently the data were analyzed reducing the number of variables in the model and analyzing the various organs separately. The classification improved, as shown in Table 1, for all the organs at both 3 h and 24 h.

### 3.2. MicroNIR Viavi

**Figure 2:**
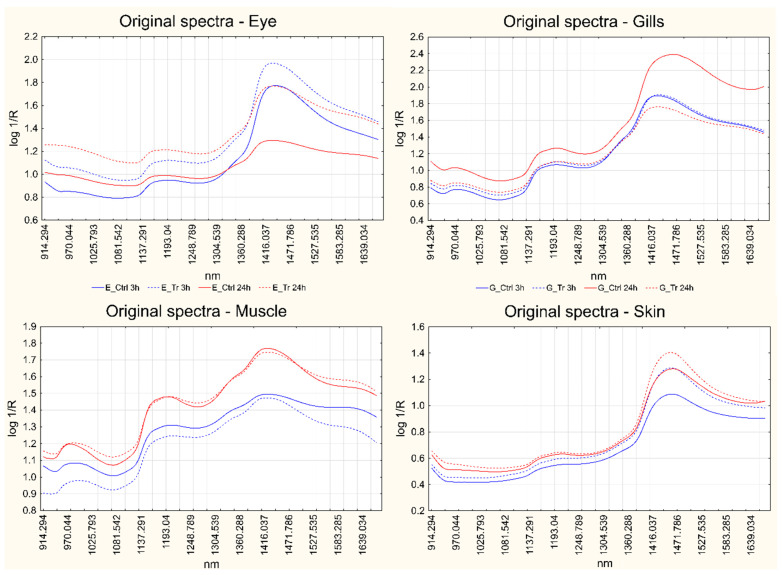
Average spectra of a control fish and its corresponding treated fish at both short- and long-term for each matrix separately: eye (**top left**); gills (**top right**); muscle (**bottom left**); skin (**bottom right**). Blue lines represent the short-term measurements, while red lines correspond to long-term treatments. Solid lines represent controls, while dotted lines represent the treated samples.

Figure 2 reports a MicroNIR spectrum obtained for each sample matrix, in the range 915–1670 nm. For each matrix, four spectra are reported: short-term control and treated spectra and long-term control and treated spectra; each spectrum is the average of three measurements recorded on the same fish. Since the study design was organized as paired comparisons, the average spectra represented belong to one control fish and its corresponding treated fish at both short- and long-term treatment. The available dataset from MicroNIR acquisitions is reported as Supplementary Table S1.

PLS-DA was applied to identify the effect played by Cafodos in the short- and long-term independently, separately for each sample matrix. Different data pre-treatments were compared as follows: Standard normal variate (SNV), first derivative and second derivative. All the models were calculated coupling PLS-DA to a variable selection procedure in backward elimination, as described in Section 2.3. The Table 2 reports the results obtained: for each matrix and separately for short- and long-term effects, the percentage of classification accuracy is given both in calibration and in cross-validation, together with the number of variables selected by the backward elimination procedure and the number of latent variables included in the models.

**Figure 3:**
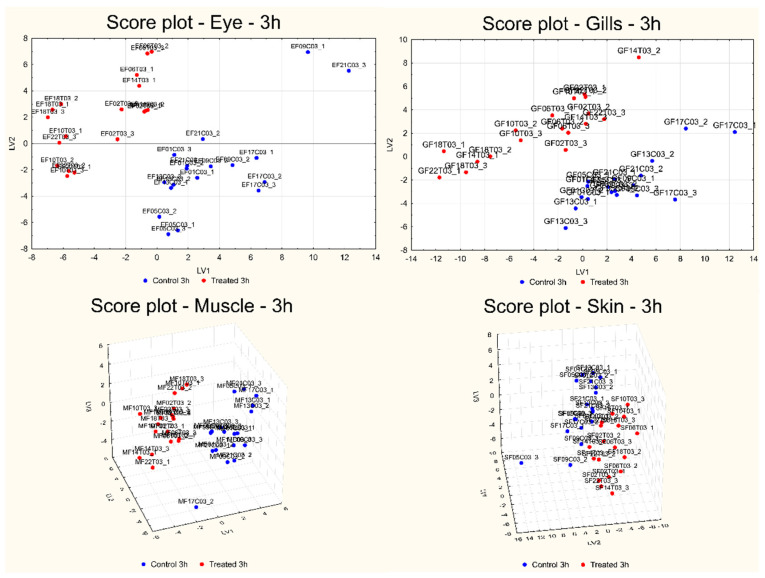
Score plot of the first two (eye and gills) or three LVs (muscle and skin) calculated for the best models obtained for each sample matrix for the short-term effect: eye (**top left**); gills (**top right**); muscle (**bottom left**); skin (**bottom right**).

**Figure 4:**
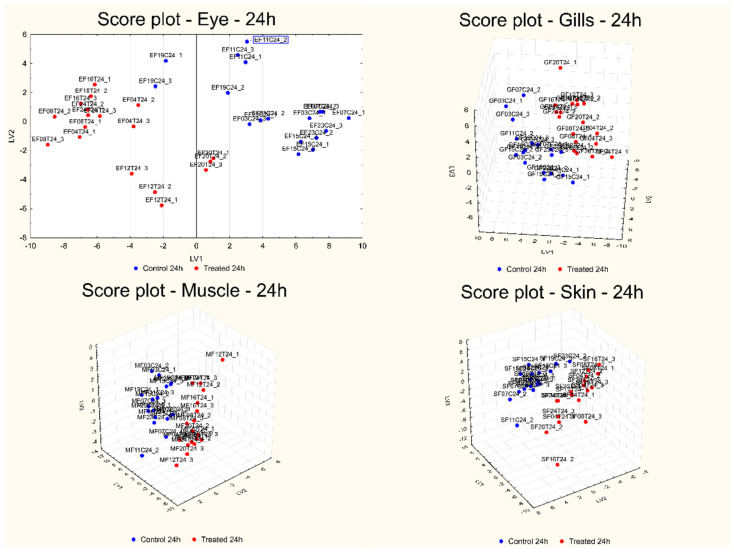
Score plot of the first two (eye) or three LVs (gills, muscle, and skin) calculated for the best models obtained for each sample matrix for the long-term effect: eye (**top left**); gills (**top right**); muscle (**bottom left**); skin (**bottom right**).

Figure 3 and Figure 4 report the score plots of the first two or three LVs calculated for the best models obtained for each sample matrix and for both short- (Figure 3) and long-term (Figure 4) effects.

**Figure 5:**
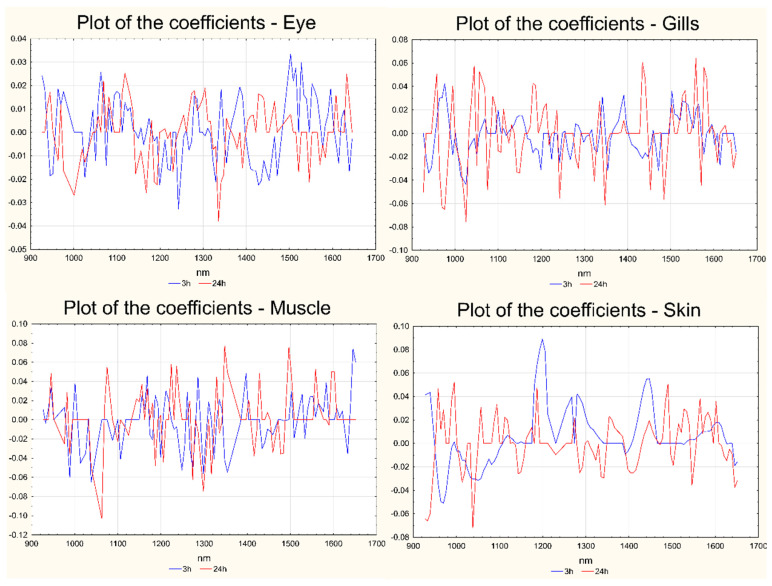
Plots of the coefficients for the best models calculated for each sample matrix in both short- and long-term: eye (**a**), gills (**b**), muscle (**c**), skin (**d**). The coefficients for short-term (3 h) are represented in blue; those for long-term (24 h) are represented in red. In all the cases the variables are reported on the x-axis and the coefficients are reported on the y-axis.

The reasons for the separation of the samples in the two classes can be explained in the corresponding plots of the coefficients, reported in Figure 5 for both short- and long-term effects and for all the sample matrices. In Figure 5, the variables are indicated on the x-axis, while the coefficients are reported on the y-axis. For each sample matrix, the coefficients calculated for the short-term model are reported in blue, while the coefficients calculated for the long-term treatment are represented in red.

## 4. Discussion

### 4.1. SCiO

In the spectra obtained from SCiO, the most characteristic bands were associated to the 3rd overtone of water (760 nm) and to the proteins (950 and 1020 nm), confirming that water is the main constituent of fish.

The data analysis followed two approaches, one based on six-variable analysis and the second on two-variable analysis. The idea was to determine in which case the best classification was obtained. Combining all data at a given time post-treatment, the samples were, in general, correctly classified (from 77% to 100%). The worst classification was obtained on muscle treated with Cafodos at 24 h.

By reducing the number of variables, great improvements were achieved in the classification of the gills, which reached 100%, and also for muscle (from 90% to 100%). For the skin, there was a slight worsening both in the treated and not treated fish.

However, in the two-variable analysis, treated and untreated for a single organ, the tool suggests that increasing the number of samples to increase the reliability of the model.

It should be noted, however, that the raw data in this SCiO version were not exportable. Therefore, the models obtained from this proprietary software cannot be processed with additional chemometric approaches.

### 4.2. MicroNIR Viavi

Looking at the spectra reported in Figure 2, in all cases, the most prominent band was located at ca. 1400 nm. This is related to the first overtone of the O-H group arising from water and chemical groups bonded with water via hydrogen bonds [24,25], thus indicating that this compound plays a major role in defining the general shape of the spectra. Even the small band at ca. 1000 nm can be attributed to water [9,26,27], although it is always much weaker than the previous one, but in some articles it was also correlated to the presence of -NH_2_ compounds [9,28]. On the other hand, the signal between 1150 and 1200 nm is generally attributed to the second overtone of C-H stretching in lipids [19,24,29,30,31]. The appearance of this band changes significantly among the different matrices; it is particularly well defined in the spectra recorded on muscles, whereas it is slightly weaker in the case of eyes and gills and it is barely noticeable in those acquired on the skin. One last weak signal arises at ca. 1330–1370 nm as a shoulder of the main water band, which could be due to C-H combination modes [19,24,32,33].

Figure 2 also points out some differences between the control and treated spectra at both short- and long-term exposures, with spectral regions that show an increasing or decreasing absorption; however, the biological variability due to the fish samples often overcomes these differences, hampering the identification of the spectral changes related to the treatment applied. This problem can be overcome by centering the data according to the day of analysis, so that each couple of paired fish samples is centered with respect to its average.

PLS-DA was, therefore, applied to identify the effect played by Cafodos in both the short- and long-term independently. The performances of the models built for each BE-PLS-DA model, with three different spectral pre-treatments (SNV, first derivative, second derivative), and for the different sample matrices independently (Table 1), show that the best results could be obtained by the second derivative, with the only exception of skin at short-term, for which the best results were obtained with the first derivative. The best models show the perfect classification of all the samples both in calibration and cross-validation.

The score plots of the first two or three LVs calculated for the best models, reported in Figure 3 (for the short-term effect) and Figure 4 (for the long-term effect), show a very good separation of the two groups of samples in all the cases. The evaluation of the reasons for the separation of the samples in the two classes in all the investigated comparisons can be carried out by looking at the corresponding plots of the coefficients, reported in Figure 5. For each sample matrix and for short- and long-term separately, the positive coefficients correspond to wavelengths with signals that, in the second derivative or first derivative (for skin at the short-term) spectra, are more intense after treatment, while the negative coefficients show the opposite behavior.

The plots of the coefficients show that the treatment with Cafodos plays an effect on all the main components of the sample (lipids, proteins, etc.) since the overall spectral region investigated is involved in the effect, i.e., shows significant positive or negative coefficients. Moreover, it is possible to notice that, for almost all the sample matrices, the effect played in the short-term is in part opposite to that played in the long-term: this effect can be identified by looking at the sign of the coefficients of the original variables on the model for the short- and long-term; a high number of variables (wavelengths) have coefficients with opposite signs for the two exposure times. This behavior is not to be considered unusual since the study design proposed here is intended to identify the spectral regions involved in two different types of effects: in the short-term we compared almost fresh fish with the Cafodos-treated counterpart (the treatment was applied for 3 h only), while in the long-term, we compared the effect of Cafodos with what should have been the effect of a “normal” storage in ice. In this last case, therefore, we did not compare fresh with Cafodos-treated samples, but rather licit vs. illicit treatment.

## 5. Conclusions

The global trend of fish and seafood consumption is increasing annually, leading to the emergence of several food frauds such as mislabeling and adulteration [1]. To counter these issues, spectroscopic techniques could be utilized for the detection of different illicit practices in food products [10,12,19]. A recent study focused on the use of Raman spectroscopy, aimed to characterize the effects of hydrogen peroxide in fish muscle and skin samples, confirmed the potential of these techniques as suitable field diagnostic tools [16]. Starting from the aforementioned study, this study aimed to verify further spectroscopic methods capable of discovering similar food fraud and in a more rapid and effective way to fill the gaps in the current available analysis technologies. By using two different handled NIR instruments, it was possible to classify fish in control or Cafodos-treated groups in both the short- and long-term.

Considering the practical applications, by using simple and rapid NIR devices, it is possible to rapidly screen fish products to evaluate Cafodos treatment without extensive and elaborate chemometric analysis. Moreover, this instrument could be used also by non-technical personnel.

In addition, the rapidity of spectroscopic measurements and the minimal sample pretreatment, not requiring the use of reagents, resulted in cheaper and safer workflows that make this method promising for widespread application, potentially even directly in the field using portable devices like those tested in the present work.

## Figures and Tables

**Table 1:** Classification results of 2-variables analysis: treated/not treated. All values are expressed in percentage.

Organ/Time	Model	F1 ^1^	Predicted NT ^2^ (%)	Predicted T ^3^ (%)
Muscle (3 h)	Normalize	0.96	100	100
Muscle (24 h)	Normalize	0.95	100	90
Gill (3 h)	Processed and Normalize	0.94	100	100
Gill (24 h)	Processed and Normalize	0.98	100	100
Skin (3 h)	(log)R and Normalize	0.83	81	91
Skin (24 h)	(log)R and Normalize	0.80	81	90

^1^ Coefficient of variation. ^2^ Not treated. ^3^ Treated with Cafodos.

**Table 2:** PLS-DA classification results in calibration and in cross-validation.

Sample Matrix	Effect	Pre-Treatment	N° Variables	N° LV ^a^	Acc% cal ^b^	Acc% cv ^c^
Eye	Short-term	Der1	86	5	100	100
		Der2	86	2	100	100
		SNV	86	5	97.22	97.22
	Long-term	Der1	86	4	97.22	97.22
		Der2	86	2	100	100
		SNV	24	5	100	100
Gills	Short-term	Der1	86	4	100	100
		Der2	86	2	100	100
		SNV	86	5	100	97.22
	Long-term	Der1	46	2	94.44	94.44
		Der2	71	5	100	100
		SNV	86	2	97.22	97.22
Muscle	Short-term	Der1	22	4	97.22	97.22
		Der2	71	5	100	100
		SNV	49	3	94.44	94.44
	Long-term	Der1	92	5	97.22	91.67
		Der2	52	4	100	100
		SNV	98	4	80.56	86.11
Skin	Short-term	Der1	86	4	100	100
		Der2	86	5	100	97.22
		SNV	23	5	91.67	88.89
	Long-term	Der1	49	5	97.22	97.22
		Der2	86	4	100	100
		SNV	52	5	100	100

^a^ Latent variables. ^b^ Accuracy of calibration. ^c^ Accuracy of cross-validation.

## Data Availability

The original contributions presented in the study are included in the article/Supplementary Materials, further inquiries can be directed to the corresponding author.

## Supplementary Materials

The following supporting information can be downloaded at: https://www.mdpi.com/article/10.3390/foods13213443/s1, Supplementary Table S1: Raw data from microNIR acquisitions.

## References

[1] Reilly A. (2018). Overview of food fraud in the fisheries sector. Fish. Aquac. Circ. No. 1165.

[2] Hungerford J.M. (2010). Scombroid poisoning: A review. Toxicon.

[3] Annunziata L., Schirone M., Campana G., De Massis M.R., Scortichini G., Visciano P. (2022). Histamine in fish and fish products: An 8-year survey. Follow up and official control activities in the Abruzzo region (Central Italy). Food Control..

[4] Bremner H.A. (2000). Toward practical definitions of quality for food science. Crit. Rev. Food Sci. Nutr..

[5] Gonçalves S., Alves V.R., Pérez S.O., Ferreira M., Daguer H., de Oliveira M.A.L., Micke G.A., Vitali L. (2020). Rapid method for the determination of citrate, phosphate and sulfite in seafood by capillary zone electrophoresis. Food Chem..

[6] Manimaran U., Shakila R.J., Shalini R., Sivaraman B., Sumathi G., Selvaganapathi R., Jeyasekaran G. (2016). Effect of additives in the shelflife extension of chilled and frozen stored Indian octopus (*Cistopus indicus*). J. Food Sci. Technol..

[7] Bello F.D., Aigotti R., Zorzi M., Giaccone V., Medana C. (2020). Multi-analyte ms based investigation in relation to the illicit treatment of fish products with hydrogen peroxide. Toxics.

[8] Ghidini S., Varrà M.O., Dall’Asta C., Badiani A., Ianieri A., Zanardi E. (2019). Rapid authentication of European sea bass (*Dicentrarchus labrax* L.) according to production method, farming system, and geographical origin by near infrared spectroscopy coupled with chemometrics. Food Chem..

[9] Ottavian M., Facco P., Fasolato L., Novelli E., Mirisola M., Perini M., Barolo M. (2012). Use of near-infrared spectroscopy for fast fraud detection in seafood: Application to the authentication of wild European sea bass (*Dicentrarchus labrax*). J. Agric. Food Chem..

[10] Esposito G., Sciuto S., Guglielmetti C., Pastorino P., Ingravalle F., Ru G., Bozzetta E.M., Acutis P.L. (2022). Discrimination between Wild and Farmed Sea Bass by Using New Spectrometry and Spectroscopy Methods. Foods.

[11] Marlard S., Doyen P., Grard T. (2019). Rapid Multiparameters Approach to Differentiate Fresh Skinless Sea Bass (*Dicentrarchus labrax*) Fillets from Frozen-Thawed Ones. J. Aquat. Food Prod. Technol..

[12] Massaro A., Stella R., Negro A., Bragolusi M., Miano B., Arcangeli G., Biancotto G., Piro R., Tata A. (2021). New strategies for the differentiation of fresh and frozen/thawed fish: A rapid and accurate non-targeted method by ambient mass spectrometry and data fusion (part A). Food Control..

[13] Benedetto A., Robotti E., Belay M.H., Ghignone A., Fabbris A., Goggi E., Cerruti S., Manfredi M., Barberis E., Peletto S. (2024). Multi-Omics Approaches for Freshness Estimation and Detection of Illicit Conservation Treatments in Sea Bass (*Dicentrarchus Labrax*): Data Fusion Applications. Int. J. Mol. Sci..

[14] Wu T.H., Bechtel P.J. (2008). Ammonia, dimethylamine, trimethylamine, and trimethylamine oxide from raw and processed fish by-products. J. Aquat. Food Prod. Technol..

[15] Uddin M., Okazaki E. (2001). Applications of Vibrational Spectroscopy to the Analysis of Fish and Other Aquatic Food Products. Handbook of Vibrational Spectroscopy.

[16] Robotti E., Belay M.H., Calà E., Benedetto A., Cerruti S., Pezzolato M., Pennisi F., Abete M.C., Marengo E., Brizio P. (2023). Identification of Illicit Conservation Treatments in Fresh Fish by Micro-Raman Spectroscopy and Chemometric Methods. Foods.

[17] Cozzolino D. (2015). Infrared spectroscopy as a versatile analytical tool for the quantitative determination of antioxidants in agricultural products, foods and plants. Antioxidants.

[18] Hassoun A., Shumilina E., Di Donato F., Foschi M., Simal-Gandara J., Biancolillo A. (2020). Emerging techniques for differentiation of fresh and frozen-thawed seafoods: Highlighting the potential of spectroscopic techniques. Molecules.

[19] Moser B., Jandric Z., Troyer C., Priemetzhofer L., Domig K.J., Jäger H., van den Oever S.P., Mayer H.K., Hann S., Zitek A. (2023). Evaluation of spectral handheld devices for freshness assessment of carp and trout fillets in relation to standard methods including non-targeted metabolomics. Food Control..

[20] Giró-Candanedo M., Cruz J., Comaposada J., Barnés-Calle C., Gou P., Fulladosa E. (2024). Differentiation between fresh and frozen-thawed mackerel fish using low-cost portable near infrared spectrometry devices. J. Food Eng..

[21] Beć K.B., Grabska J., Huck C.W. (2020). Principles and Applications of Miniaturized Near-Infrared (NIR) Spectrometers. Chem. A Eur. J..

[22] Oussama A., Elabadi F., Platikanov S., Kzaiber F., Tauler R. (2012). Detection of olive oil adulteration using FT-IR spectroscopy nd PLS with variable importance of projection (VIP) scores, JAOCS. J. Am. Oil Chem. Soc..

[23] Ballabio D., Consonni V. (2013). Classification tools in chemistry. Part 1: Linear models. PLS-DA. Anal. Methods.

[24] Zhou J., Wu X., Chen Z., You J., Xiong S. (2019). Evaluation of freshness in freshwater fish based on near infrared reflectance spectroscopy and chemometrics. LWT.

[25] Lan W., Liu J., Hu X., Xiao L., Sun X., Xie J. (2021). Evaluation of quality changes in big-eye tuna (*Thunnus obesus*) based on near-infrared reflectance spectroscopy (NIRS) and low field nuclear magnetic resonance (LF-NMR). J. Food Process Eng..

[26] Wang X., Shan J., Han S., Zhao J., Zhang Y. (2019). Optimization of fish quality by evaluation of total volatile basic nitrogen (TVB-N) and texture profile analysis (TPA) by near-infrared (NIR) hyperspectral imaging. Anal. Lett..

[27] Sivertsen A.H., Kimiya t., Heia K. (2011). Automatic freshness assessment of cod (*Gadus morhua*) fillets by Vis/Nir spectroscopy. J. Food Eng..

[28] Park B., Chen Y.R., Hruschka W.R., Shackelford S.D., Koohmaraie M. (2001). Principal component regression of near–infrared reflectance spectra for beef tenderness prediction. Trans. ASAE.

[29] Ding R., Huang X., Han F., Dai H., Teye E., Xu F. (2014). Rapid and nondestructive evaluation of fish freshness by near infrared reflectance spectroscopy combined with chemometrics analysis. Anal. Methods.

[30] Zhao H., Guo B., Wei Y., Zhang B. (2014). Effects of grown origin, genotype, harvest year, and their interactions of wheat kernels on near infrared spectral fingerprints for geographical traceability. Food Chem..

[31] Teye E., Huang X., Lei W., Dai H. (2014). Feasibility study on the use of Fourier transform near-infrared spectroscopy together with chemometrics to discriminate and quantify adulteration in cocoa beans. Food Res. Int..

[32] Kamruzzaman M., Sun D.W., ElMasry G., Allen P. (2013). Fast detection and visualization of minced lamb meat adulteration using NIR hyperspectral imaging and multivariate image analysis. Talanta.

[33] Sinelli N., Casale M., Di Egidio V., Oliveri P., Bassi D., Tura D., Casiraghi E. (2010). Varietal discrimination of extra virgin olive oils by near and mid infrared spectroscopy. Food Res. Int..

